# Phase I/II study of gemcitabine plus mitoxantrone as salvage chemotherapy in metastatic breast cancer

**DOI:** 10.1038/sj.bjc.6600780

**Published:** 2003-02-18

**Authors:** V Lorusso, E Crucitta, N Silvestris, A Catino, L Caporusso, A Mazzei, M Guida, A Latorre, D Sambiasi, C D'Amico, F Schittulli, P Calabrese, M De Lena

**Affiliations:** 1 Operative Unit of Medical Oncology, Oncology Institute, Via Amendola 209, Bari, Italy; 2 Operative Unit of Breast Cancer Surgery, Oncology Institute, Via Amendola 209, Bari, Italy; 3 Operative Unit of Cardiology, Oncology Institute, Via Amendola 209, Bari, Italy

**Keywords:** breast cancer, metastatic disease, gemcitabine, mitoxantrone, phase I/II study, chemotherapy

## Abstract

The purpose of this study was to determine the maximum-tolerated dose of gemcitabine plus mitoxantrone in women with metastatic breast cancer (MBC) and to evaluate activity and toxicity of this combination in a phase II trial. Sixty-three patients with MBC, previously treated with chemotherapy including anthracycline and/or taxanes, were treated with mitoxantrone 10 or 12 mg m^−2^ intravenously on day 1 plus gemcitabine in escalating doses from 600 to 1200 mg m^−2^ intravenously on days 1 and 8, every 3 weeks. In phase I, on 23 patients entered on study, dose-limiting toxicity occurred at the dosage of 1200 mg m^−2^ gemcitabine and 10 mg m^−2^ mitoxantrone, with three out of five patients developing grade 4 neutropenia. In phase II, with gemcitabine administered at 1000 mg m^−2^ and mitoxantrone at 10 mg m^−2^, 12 (30%) out of 40 assessable patients responded, even if no complete response was obtained. Moreover, stable disease was observed in eight (20%) patients. The median time to treatment failure was 22 weeks (range, 2–33), and median survival was 42 weeks (range, 2–92). Grade 3 and 4 neutropenia were observed in 12 (30%) and one (2.5%) cases respectively; grade 3 thrombocytopenia was observed in two patients (5%), grade 2 mucositis in two patients (5%), grade 3 anaemia in two patients (5%), grade 3 alopecia in one patient (2.5%) and asymptomatic cardiotoxicity in three patients (8%), respectively. In conclusion, the doses of 10 mg m^−2^ (day 1) for mitoxantrone and 1000 mg m^−2^ for gemcitabine (days 1–8) every 3 weeks resulted active and safe in MBC. Further investigations in less heavily pretreated patients are warranted.

Breast cancer is the most common malignancy affecting women in USA and in Europe with an incidence rate of 21 and 28%, respectively, among all causes of cancer (Black *et al*, 1997; Greenlee *et al*, 2000). Despite the progress achieved in screening and management of the early stages of the disease, 40–60% of subjects with this malignancy will develop metastases and will ultimately die of the disease. A number of cytotoxic drugs have shown activity in these patients, but actually combinations of taxanes and anthracyclines are the most extensively utilised as first-line treatment (Perez, 1999). In fact, with these regimens an overall response rate (ORR) of 40–80% with a complete response (CR) of less than 20% is currently achievable in not pretreated patients (Dieras, 1998; De Lena *et al*, 2000; Pagani *et al*, 2000). However, the increasing use of anthracyclines and taxanes in the adjuvant setting has led to a limitation of the role of these drugs in relapsed patients (Hortobagy and Buzdar, 1993; Early Breast Cancer Trialists' Collaborative Group, 1998; Goldhrisch *et al*, 2000). As a consequence, new cytotoxic drugs and new combination therapies are needed as second-line treatment.

Mitoxantrone hydrochloride is an anthracene derivative whose effectiveness in metastatic breast carcinoma has been well documented. In fact, this drug as single agent is able to achieve 30–36% responses in first-line chemotherapy and about 20% responses in second line. Its toxicity includes limited vomiting, oral mucositis, hair loss and cardiotoxicity (Smith *et al*, 1983; Mouridsen *et al*, 1985; Robertson *et al*, 1989; Brufman *et al*, 1993).

Gemcitabine hydrochloride, a novel nucleoside analogue, offers proven activity against a range of solid tumours (Gatzemeier *et al*, 1996; Moore, 1996; Shapiro *et al*, 1996; Stadler *et al*, 1997). In particular, in metastatic breast cancer, gemcitabine alone yielded response rates of 25–46% in the first phase II studies (Hertel *et al*, 1990; Carmichael *et al*, 1995; Blackstein *et al*, 1997; Spielmann *et al*, 1997; Possinger *et al*, 1999). Haematological and nonhaemato-logical side effects were mild, with dose reductions, treatment delays or withdrawals only seldom reported. Moreover, also the first phase II studies of this cytotoxic drug in combination with anthracyclines, docetaxel or vinorelbine demonstrated a good toxicity profile and promising results (Mavroudis *et al*, 1999; Nicolaides *et al*, 2000; Pérez-Manga *et al*, 2000). The rationale for the combination of gemcitabine plus mitoxantrone was the therapeutic efficacy, the low systemic toxicity and the high tolerability of both drugs. Thus, the aim of this phase I/II study was to evaluate the maximum tolerated dose (MTD), the activity (response, time to treatment failure, survival) and toxicity profile of this combination in patients with stage IV breast cancer patients previously treated with chemotherapy regimens including taxanes or anthracyclines.

## PATIENTS AND METHODS

Patients with histologically proven, measurable locally advanced or metastatic breast cancer, refractory or resistant to hormone therapy, who had previously received chemotherapy including anthracyclines or taxanes, were eligible for this study. However, patients may have received as maximum anthracycline dosage a cumulative dose of 300 or 540 mg m^−2^ of doxorubicin and epidoxorubicin, respectively. The other eligibility criteria were: age between 18 and 70 years; Eastern Cooperative Oncology Group (ECOG) performance status (PS) ⩽2; expected survival of ⩾3 months; good bone marrow function (absolute granulocyte count ⩾4000 ml^−1^, platelet count ⩾100.000 ml^−1^, and haemoglobin ⩾11 g dl^−1^); adequate hepatic and renal function, including AST, ALT, bilirubin, alkaline phosphatase and serum creatinine values ⩽1.25 times the upper limit of normal; no severe uncontrolled comorbidity and no other second malignancy. Patients with central nervous system metastasis were eligible for this study if they were asymptomatic and had received whole-brain irradiation. Written informed consent was obtained from all participating patients before study entry. The pretreatment evaluation included medical history and physical examination with tumour measurements: chest X-ray, complete blood cell count, serum chemistries, liver function tests; staging studies to define the extent of metastatic disease which included abdominal ultrasound, thoracic and/or abdominal computed tomography (CT), bone scan, as indicated by clinical or laboratory examination.

### Treatment plan

Patients received 10 or 12 mg m^−2^ mitoxantrone (Novantrone®) diluted in 100 ml of normal saline over a 30-min intravenous infusion on day 1. Gemcitabine was administered immediately after mitoxantrone diluted in 250 ml of normal saline in 30 min on days 1 and 8. Using the modified Fibonacci schema (Von Hoff *et al*, 1984), gemcitabine doses were escalated in subsequent steps, with a minimum of three patients entered and maintained per dose level throughout their therapy. The starting dose of 600 mg m^−2^ gemcitabine was escalated in 200 mg m^−2^ increments until dose-limiting toxicity (DLT) developed in three of five patients treated at a given dose level. After the DLT of gemcitabine was reached, a mitoxantrone dose level of 12 mg m^−2^ was tested in combination with the lowest dosage of gemcitabine in order to obtain a better balance in dose intensity between the drugs. The chemotherapy cycle was administered every 3 weeks to a maximum total of eight cycles for patients with CR, partial response (PR), or stable disease (SD). With regard to drug supply, mitoxantrone (Novantrone®) was currently available in Italy for the treatment of advanced breast cancer, whereas gemcitabine hydrochloride (Gemzar®) was kindly provided by Eli Lilly and Company, Indianapolis, USA.

### Toxicity and evaluation of response

Clinical monitoring was performed twice a week with a complete blood cell count. Toxicity was scored using the World Health Organization (WHO) criteria (WHO, 1979). At least three patients were treated at each dose level. To determine the MTD of this combination, toxicity was assessed if any of these events occurred: (1) grade 4 thrombocytopenia or grade 4 neutropenia; (2) grade 2 renal or liver dysfunction; (3) any other nonhaematological, nonhepatic and nonrenal grade 3 toxicity excluding alopecia; and (4) impossibility of delivering full dose of gemcitabine on day 8 because of grade 2–4 neutropenia or thrombocytopenia. If any of the side effects reported above was observed in one of three patients, another patient entered the study at the same dose level. If DLT was observed in two out of three or four, a fifth patient was added. If DLT was observed in zero out of three, one out of four or two out of five patients, respectively, at a single-dose level, we escalated the dose. If we observed DLT in three out of three, four or five patients, previous dose level was the MTD recommended for the phase II trial (best of five schedule). Antitumour activity was evaluated every two courses (i.e. 6 weeks) on all measurable lesions. All patients were scheduled for at least two cycles in order to be eligible for assessment of tumour response. In patients with tumour responses or SD, the treatment was continued to a maximum of eight cycles. Tumour response was classified according to WHO criteria and documented by two investigations at least 6 weeks apart.

CR was defined as the disappearance of all clinical evidence of active tumour with complete reossification of bone lesion and the absence of any disease-related symptoms for a minimum of 4 weeks. PR was defined as a ⩾50% reduction in the sum of the products of the perpendicular diameters of all measurable lesions, in absence of any new lesion for at least 4 weeks. In the case of multiple metastatic sites of disease, the largest masses (up to five) were considered as the index lesions. Minor response (MR) was defined as a decrease in tumour size of less than 50% but more than 25%, for at least 4 weeks. SD was defined as a less than 25% decrease or a less than 25% increase in tumour size for at least 4 weeks. Progressive disease (PD) was defined as the unequivocal appearance of any new lesion or an increase of ⩾25% in the sum of the perpendicular diameters of any measured lesion or in the estimated size of a not measurable lesion. MR, SD and PD were considered as treatment failures. The Kaplan–Meier method was used to analyse the time to progression (TTP) and overall survival (OS). The confidence intervals (CIs) for the response rates were calculated using the method described by Simon (1986). The sequential two steps statistical test of Gehan (1961) was used to define the number of patients required to detect activity of the treatment. Cardiotoxicity was carefully monitored. In fact, all patients before study entry were submitted to physical examination by a cardiologist, EKG and basal echocardiogram with measurement of left ventricular ejection fraction (LVEF). Patients with less than 50% LVEF were excluded from the study. Echocardiogram was performed every other cycle and patients were excluded from study continuation if, at any time, their LVEF decreased by 10% below the initial value, even if they were still asymptomatic.

## RESULTS

From October 1997 to January 2000, 63 women with a median age of 52 (range 34–69) years, were enrolled into this phase I/II study: 23 patients in the phase I study (three patients at dose levels 1 and five at dose levels 2, 3, 4 and 5, respectively) and 40 in the subsequent phase II study. The patient characteristics are shown in Table 1

**Table 1:** Phases I and II – patient characteristics

	**Phase I**	**Phase II**
	**No. of patients**	**No. of patients**
	**(*N*=23)**	**(*N*=40)**
Enrolled	23	40
Median age (years)	52 (range 34–69)	55 (range 37–73)
*Performance status (ECOG)*^a^		
0–1	20	40
2	3	
*Estrogen receptors*		
Positive	8	22
Negative	15	7
Unknown		11
*Menopausal status*		
Pre	6	13
Post	17	27
*Median disease-free interval (months)*		
0–12	6	10
12–24	12	21
24	5	9
*Previous anthracyclines*	23	40
Adjuvant setting	5	9
Advanced disease setting	18	31
*Previous taxanes*	16	25
*Previous chemotherapy lines*		
One	11	18
Two	6	9
Three	6	13
*Metastatic sites*		
Viscera	11	19
Bone	6	8
Nodes and soft tissues	6	13

a Eastern Cooperative Oncology Group.

. It is noteworthy that all patients were previously submitted to anthracyclines. However, five out of 23 (22%) and nine out of 40 (22.5%) patients of the phase I and II respectively, had received anthracyclines in adjuvant setting. Moreover, overall 41 out of 63 (65%) patients had received also taxanes and 34 out of 63 (54%) had received more than one line of chemotherapy (Table 1).

### Dose escalation (phase I)

Twenty-three patients were enrolled into the phase I portion of the study in five cohorts (Table 2

**Table 2:** Number of patients with WHO grade 3 or 4 toxicities and response by dose level (no. of patients=23)

	**Dose level**				
	**1**	**2**	**3**	**4**	**5**
Patients treated	3	5	5	5	5
Gemcitabine (mg m^−2^)	600	800	1000	1200	600
Mitoxantrone (mg m^−2^)	10	10	10	10	12
*Toxicity*					
Neutropenia (DLT)	—	2	2	3	3
Thrombocytopenia	—	—	1	—	2
Anaemia	—	—	—	—	1
Vomiting	—	—	—	1	—

WHO=World Health Organization; DLT=dose-limiting toxicities.

). The dosage of mitoxantrone was 10 mg m^−2^ in dose levels 1–4 and was escalated to 12 mg m^−2^ in the dose level 5. The dosage of gemcitabine was escalated by 200 mg m^−2^ starting from 600 mg m^−2^ (dose level 1) to 1200 mg m^−2^ (dose level 4). Moreover, 600 mg m^−2^ of gemcitabine were combined with mitoxantrone 12 mg m^−2^ in dose level 5. No DLT was observed in the first three patients treated at dose level 1 (gemcitabine 600 mg m^−2^). At dose level 2 (gemcitabine 800 mg m^−2^) as well as at dose level 3 (gemcitabine 1000 mg m^−2^) two out of five patients had grade 3 neutropenia on day 8 not allowing drug administration (one patient of the latter group showed also grade 4 thrombocytopenia). At dose level 3, two of the five patients treated showed a DLT (one complained neutropenia and one neutropenia plus thrombocytopenia). Neutropenia, as DLT, occurred at dose level 4 (gemcitabine 1200 mg m^−2^). In fact, at this dose level, three patients had day 8 dose omission because of grade 3 neutropenia and one of them developed also grade 4 febrile neutropenia (lasting for more than 4 days). Subsequently, we raised mitoxantrone dose to 12 mg m^−2^ and combined this drug with 600 mg m^−2^ gemcitabine. At these dosages, three out of five patients had grade 4 neutropenia not allowing further dose escalation. Thus, the recommended dose level for the phase II study was gemcitabine 1000 mg m^−2^ and mitoxantrone 10 mg m^−2^. Overall, grades 3 and 4 neutropenia were observed in four out of 23 (17%) and six out of 23 (26%) patients, respectively. Moreover, neutropenia (febrile in four cases) frequently occurred between days 11 and 16 of the cycle. The median time to recover from neutropenia was 9 days (range 3–16). Haematological growth factors as granulocyte colony stimulating factors (G-CSF) were given only to patients with febrile neutropenia. Grade 3 or 4 thrombocytopenia were less frequent than neutropenia, occurring only in two (9%) and one (4%) patients, respectively. A severe anaemia, requiring red blood cell transfusion, occurred in one patient affected by liver metastasis and peritoneal carcinosis with haematic ascites. Nonhaematological toxicity was negligible. One patient experienced severe vomiting on day 1 of gemcitabine therapy. Liver transaminase elevation, alopecia, fever and cutaneous rash, all of grade 1–2, were observed each in one patient, respectively. With regard to cardiotoxicity, two patients previously treated with the maximum dose of anthracyclines showed a reduction of more than 10% of LVEF as compared to basal value without any clinical symptom of heart failure.

Of the 23 enrolled patients, three discontinued treatment after the first or second cycle (one due to early progression, one due to severe toxicity and one due to refusal to continue), and were therefore considered treatment failures. Upon disease reevaluation after the second cycle, there were eight partial responses. At the fourth cycle evaluation, two out of the eight partial responders demonstrated CR, while the other six patients remained partial responders, for an ORR of 35% (95% CI: 21–53%). Responses were observed at all dose levels. Three (13%) patients had SD and 12 (52%) patients had treatment failure. The five patients who had prior anthracycline as adjuvant therapy responded. Conversely, only three (16.5%) out of 18 patients treated with anthracyclines for advanced disease showed tumour regression when treated with the combination of gemcitabine plus mitoxantrone.

### Phase II

Forty patients were enrolled into this trial and all were assessable for response and toxicity. Patient characteristics are reported in Table 1. With regard to the site of disease, of the 40 assessable women, 14 had liver metastasis and, of these, three had liver metastasis alone, one liver metastasis plus ascites and 10 liver, lung and bone metastasis. Moreover, eight patients had bone, five lung and 13 soft tissues as the only site of disease respectively. All the 40 assessable patients were previously treated with anthracyclines, and 18 also with taxanes. With regard to previous chemotherapy, 18, nine and 13 patients have been treated, respectively, with one, two or three chemotherapy regimens before study entry. Nine out of 18 patients (50%) treated with one chemotherapy regimen only had received anthracyclines in adjuvant setting. For what concerns clinical response, no patient showed CR, while PR was observed in 12 patients (30%). Moreover, SD and PD were documented in eight (20%) and 20 patients (50%), respectively (Table 3

**Table 3:** Phase II results

**Response**	**No. of patients**	**%**
Partial response	12	30
Stable disease	8	20
Progression	20	50
		
Total	40	100

). Responses were observed in six patients with soft-tissue disease, two patients with liver metastases and four patients with lung, liver and bone disease. Median time to treatment failure was 22 (range, 2–29) weeks, median survival was 42 (range, 2–92) weeks and 1-year OS was 22.5%. No statistically significant differences were found in response rates or in response duration with respect to PS, menopausal or hormone receptor status, number of metastatic sites, dominant metastatic site and disease-free survival. The average dose intensity administered was 78% for gemcitabine and 89% for mitoxantrone. All the 40 assessable patients received at least one cycle of therapy with a median number of six cycles (range, 1–10). Of the 187 cycles administered during this trial, 17 (9%) were delayed: 15 because of haematological toxicity and the remaining two for nonmedical reasons. Moreover, in 15 out of 187 (8%) cycles, doses were reduced as a consequence of haematological toxicity and G-CSF was administered in 30 out of 187 cycles (16%).

Neutropenia resulted in the most common haematological toxicity observed with a grade 3 and 4 occurring in 13 (32.5%) and one (2.5%) case, respectively. Grade 3 thrombocytopenia was observed only in three patients (7.5%), even if no platelet transfusions were needed. In order to deliver drug at a dose intensity as close as possible to the planned, G-CSF was administered in 11 patients with grade 3 and in one patient with grade 4 neutropenia respectively. Other haematological and nonhaematological toxicity occurred in few patients. In particular, grade 3 anaemia and grade 3 mucositis were observed in two cases (5%), alopecia in one case (2.5%). With regard to cardiac toxicity, three patients showed a decrease of LVEF of more than 10% below the initial value and discontinued mitoxantrone after two, four and five cycles, respectively. These three patients had received a cumulative dose of epidoxorubicin equal or superior to 450 mg m^−2^ (Table 4

**Table 4:** Cardiac toxicities in patients who received mitoxantrone

		**LVEF %**			
**Patients no. 5**	**Cumulative dose (mg m^−2^)**	**Before**	**After**	**Prior anthracyclines (mg m^−2^)**	**Symptom and sign**
(1) Phase I	45	55	40	Doxo 300	Asymptomatic
(2) Phase I	65	55	45	Doxo 280	Asymptomatic
(3) Phase II	20	55	43	Epi 480	Asymptomatic
(4) Phase II	40	50	37	Epi 480	Tachycardia
(5) Phase II	50	60	45	Epi 450	Asymptomatic

Epi=epidoxorubicin, Doxo=doxorubicin.

).

## DISCUSSION

This study shows that the combination of mitoxantrone (10 mg m^−2^ d.1) plus gemcitabine (1000 mg m^−2^ d.1–8), every 3 weeks, is active and safe in patients affected by advanced breast cancer previously treated with chemotherapy including anthracyclines or taxanes. In fact, these dosages were chosen based on the phase I study results, although two of the five patients treated at this dose level showed neutropenia or leuko-thrombocytopenia. However, in patients subsequently treated in the phase II study, this dosage resulted very manageable.

Gemcitabine is a novel drug in the treatment of breast cancer. The first phase II study evaluating this drug in the treatment of locally advanced or metastatic breast cancer was published by Carmichael *et al* (1995). Forty-four patients with one prior chemotherapy regimen received gemcitabine 800 mg m^−2^ on days 1, 8, and 15 of a 28-day cycle. Of the 40 evaluable patients, three (7.5%) achieved CR and seven (17.5%) PR, with an ORR of 25%. Responses were observed in all measurable metastatic sites (soft tissue, liver and lung). Median time to response and survival were 1.9 and 11.5 months, respectively. Similar results were reported with higher doses of gemcitabine in two other phase II studies on metastatic breast cancer (Blackstein *et al*, 1997; Spielmann *et al*, 1997). In particular, Spielmann *et al* (1997) evaluated 43 women previously treated with anthracyclines and achieved an ORR of 28% (95% CI 15–44%), with a dose of 1200 mg m^−2^ given at the same schedule as above. More recently, with a lower dosage (1000 mg m^−2^) and the same schedule, Possinger *et al* (1999) reported a 14.3% response, with minimal toxicity, in 42 not previously treated patients with prevalent visceral disease. However, despite the modest activity of gemcitabine as single agent in metastatic breast cancer, its favourable toxicity profile makes it an ideal candidate for combination chemotherapy. In fact, Pérez-Manga *et al* (2000) treated 42 patients with the combination of gemcitabine and doxorubicin and obtained three complete and 20 partial responses, with an ORR of 55%. Moreover, Mavroudis *et al* (1999) with the combination of docetaxel and gemcitabine plus G-CSF, observed 54% response in anthracycline-pretreated patients. Nicolaides *et al* (2000) treated 31 patients, who had shown progression after a first-line taxanes-based chemotherapy with the combination of gemcitabine 1000 mg m^−2^ days 1–8 and vinorelbine 30 mg m^−2^ days 1–8. Of 27 evaluable patients, one (4%) achieved complete remission and five (18%) partial remission.

Mitoxantrone is an active, well-tolerated drug in the treatment of advanced breast cancer. A number of studies have demonstrated that this drug has an activity comparable to anthracyclines, but it is much less toxic (Bennett *et al*, 1988; Henderson *et al*, 1989). Given as a single agent, mitoxantrone is able to achieve up to 27% response in patients previously treated with chemotherapy (Brufman *et al*, 1993). Moreover, an incomplete cross resistance with doxorubicin has been suggested. In fact, Smith *et al* (1983), with mitoxantrone as single agent, reported three out of nine responses in patients refractory to doxorubicin. Furthermore, the rationale for the use of a mitoxantrone combination after anthracyclines is given by the results reported by Panagos *et al* (Panagos, 1997) and Susnjar *et al* (1999). In fact, the former, using a combination of mitoxantrone and paclitaxel, observed seven partial remissions plus six minor responses or disease stabilisation out of 14 anthracycline pretreated patients, and the latter, using a combination of mitoxantrone plus folinic acid and fluorouracil, reported a high percentage of response in doxorubicin-resistant breast cancer.

It is noteworthy that, although the potential high risk of cardiac toxicity from mitoxantrone, in patients previously treated with anthracyclines, cardiac dysfunction was only seldom reported in the above mentioned studies. Also in the present study, five out of 63 patients (8%) showed a drop of more than 10% of LVEF as compared to basal value, without any sign or symptom of cardiac toxicity. It is likely that the reason for the low incidence of clinical cardiotoxicity in our study is because cardiac function was closely monitored, thus allowing patients to discontinue treatment prior to clinical evidence of cardiac toxicity.

In conclusion, the high activity of mitoxantrone in breast cancer and the suggested activity with low toxicity profile for gemcitabine represented the rationale of our study, whose aims were the definition of the MTD and the assessment of efficacy of this combination. The dose of gemcitabine of 1000 mg m^−2^ on days 1 and 8 plus mitoxantrone 10 mg m^−2^ on day 1 of a 3-week schedule, obtained 30% partial response in 40 patients. Our data show that the administration of these two drugs was associated with a manageable haematological toxicity and negligible nonhaematological side effects. Also the fearful cardiac dysfunction that might be expected in high percentage in these patients, because of their prior anthracycline treatment, was a minor problem. Further studies in less heavily pretreated patients are warranted with this combination.
